# LINC00922 acts as a novel oncogene in gastric cancer

**DOI:** 10.1186/s12957-022-02569-3

**Published:** 2022-04-15

**Authors:** Zeyu Ji, Yuping Qiu, Qingchun Cai, Chunfang Xu

**Affiliations:** 1 Department of Digestive Medicine, The First Affiliated Hospital of Soochow University, Suzhou, 215006 Jiangsu China; 2 Yancheng First Hospital, Affiliated Hospital of Nanjing University Medical School, Nanjing, China; 3 The First People’s Hospital of Yancheng, Yancheng, 224006 Jiangsu China; 4 81st Hospital of the Chinese People’s Liberation Army, Nanjing, 210002 Jiangsu China

**Keywords:** LINC00922, miR-204-5p, HMGA2, Gastric cancer

## Abstract

**Background:**

Long non-coding RNAs (lncRNAs) have been discovered to participate in various cancer developments. However, the biological function of lncRNAs associated with gastric cancer (GC) has not been fully elucidated.

**Methods:**

Quantitative RT-PCR (qRT-PCR) assay was performed to measure lncRNAs, microRNAs (miRNAs) and message RNA (mRNA) expression. Cell Counter Kit-8 (CCK-8), clone formation, wound healing, and transwell assays were performed to investigate cell proliferation, migration, invasion, and apoptosis. Fluorescence in situ hybridization (FISH) assay was used to analyze LINC00922 in either the cytoplasm or nucleus. The potential binding among lncRNA, miRNA, and mRNA was evidenced by bioinformatics, luciferase reporter assay. Mouse-xenograft experiments were used to explore the tumorigenesis in vivo.

**Results:**

LINC00922 was upregulated in GC, and high LINC00922 expression was associated with poor prognosis. Inhibition of LINC00922 suppressed GC cell proliferation, migration, invasion, and activated cell apoptosis in vitro and inhibited tumorigenesis in vivo. Besides, LINC00922 was markedly located in the cytoplasm. The mechanistic analysis demonstrated that LINC00922 acted as a sponge of miR-204-5p, thereby inhibiting the expression of the target gene-High Mobility Group AT-hook 2 (HMGA2).

**Conclusion:**

LINC00922 accelerated the progression of GC by miR-204-5p/HMGA2 axis. These findings support LINC00922 may be a promising option for the diagnosis and therapy of GC.

**Supplementary Information:**

The online version contains supplementary material available at 10.1186/s12957-022-02569-3.

## Introduction

Gastric cancer (GC) is the third leading cause of cancer death worldwide [1]. Although advances in chemotherapy, radiotherapy and targeted therapy have partly improved clinical outcomes, the prognosis of advanced GC remains poor [2, 3]. Early GC has no specific symptoms, leading to late diagnosis and limited clinical treatment [4]. Hence, identifying molecules of GC is vital for the development of diagnostic markers and new effective treatment methods in GC.

Long non-coding RNAs (lncRNAs) are a kind of functional transcript with a length of more than 200 nucleotides [5]. The disorder of lncRNAs expression is deeply implicated in the occurrence and development of numerous human tumor diseases including GC [6–12]. For instance, LncRNA SNHG7 is considered a vital oncogene in GC [8, 9]. Besides, high expression of LINC00483 promotes cell viability, migration and invasion [12, 13]. Therefore, lncRNAs are potential molecular markers and therapeutic targets for tumor diagnosis and prognosis [14]. Unfortunately, the roles of a large number of lncRNAs associated with GC have not been elucidated. Although LINC00922 has been proven to modulate osteosarcoma [15], lung cancer [16], breast cancer [17], and its biological function in GC remain unclear.

Competing endogenous RNAs (ceRNAs) have attracted much attention in academic circles in recent years [18–21]. As ceRNAs, lncRNAs exist in the cytoplasm and have specific binding sites of microRNAs (miRNAs) [22]. MiRNAs with 18–25 nucleotides bind to the 3′-untranslated region (3′-UTR) complementary sequence of mRNA [23]. For instance, LINC01234 plays a tumor-promoting role in GC by regulating the level of CBFB through sponge miR-204-5p [24].

Here, we purposed to shed light on the molecular mechanism of LINC00922 in GC. We identified LINC00922 contributed to the progression of GC by acting as a ceRNA of miR-204-5p, thereby inhibiting the level of target gene-High Mobility Group AT-hook 2 (HMGA2).

## Materials and methods

### Tissues collection

Forty pairs of GC tissues and the corresponding non-tumor tissues were collected from The First Affiliated Hospital of Soochow University between April 2018 and September 2018. The characteristics of participants were listed in Supplementary Table 1. The consent agreement was signed and approved by the Ethics Committee of The First Affiliated Hospital of Soochow University.

### Cell culture

GES-1, MKN-45, HGC-27, AGS, and NCI-N87 were purchased from American Type Culture Collection (ATCC, USA). Cells were seeded in RPMI 1640 medium (Invitrogen, USA) with 10% fetal bovine serum (FBS, Invitrogen, USA) at 37 °C with 5% CO_2_. For in vitro transfection, short hairpin RNA against LINC00922 (sh-LINC00922), sh-HMGA2, miR-204-5p mimic and miR-204-5p inhibitor were synthesized by GeneScript (Shanghai, China) and sequences were listed in Supplementary Table 2. When cells were upon to 70% confluent, 50 nM of the corresponding sequences were transfected by Lipofectamine 2000 (Invitrogen, USA) or RNAiMax transfection (Invitrogen, USA).

### Quantitative RT-PCR (qRT-PCR) assay

Total RNA was isolated using Trizol reagent (Invitrogen, USA) and evaluated by NanoDrop (Thermo, USA). TaqMan miRNA probes and SYBR Green dye (Ambion, USA) were used to quantify miRNA and mRNA. qRT-PCR was performed using SYBR Premix Ex TaqII (Takara, Japan). Fold-changes were calculated by 2^−ΔΔCT^ method with U6 and GAPDH as internal reference. Primers were listed in Supplemental Table 3.

### Cell viability assay

Cell viability was determined by Cell Counter Kit-8 (CCK-8, Beyotime, China). In brief, cells (5 × 10^4^) were seeded into 96-well plates. After 24 h, 48 h, 72 h, and 96 h, 10 μL of CCK-8 reagent was added to cells at 37 °C for 2 h. The optical density was detected at 450 nm.

### Clone formation assay

Cells (5 × 10^3^) were seeded into 6-well plates for 2 weeks. Then the cells were fixed in methanol (Sigma, USA) for 15 min and stained with crystal violet (Sigma, USA) for 20 min. Cell colonies were observed under a light microscope (Olympus, Japan).

### Wound healing assay

In brief, cells (5 × 10^5^) were seeded into 6-well plates. When the cells have grown and fused, a sterile tube was used to scratch the cells. 0 h and 24 h after scratches, the width of the scratch was calculated by taking photos with a light microscope (Olympus, Japan).

### Transwell assay

For cell migration, cells (5 × 10^4^) were seeded into the upper transwell chamber with serum-free medium (Invitrogen, USA). For cell invasion, cells were seeded into the upper transwell chamber and pre-treated with Matrigel (BD biosciences, USA). The culture medium with 20% FBS was added into the lower transwell chamber. After 24 h, the migration cells were fixed in methanol (Sigma, USA) for 15 min and crystal violet (Sigma, USA) for 20 min. The number of cell migration and invasion was observed under a light microscope (Olympus, Japan).

### Flow cytometry assay

Cells were harvested and resuspended in Binding Buffer. Then, 5 μL Annexin V-FITC and 10 μL PI (Beyotime, China) were added to 500 μL cells for 10 min. The incubated cells were protected from light. The apoptotic rate was detected immediately by a flow cytometer (BD Biosciences, USA).

### Fluorescence in situ hybridization (FISH) assay

The probe signal was detected using FISH kit (GeneScript, China). In brief, cells were fixed in 4% paraformaldehyde for 15 min and hybridized with LINC00922 probe for 30 min. Then, the nuclei were stained with DAPI. The images were observed by a fluorescence microscope (Nikon, Japan). The sequence for LINC00922 probe was 5′-CCTCCACCACCTCCTCTTCTG-3′.

### Dual-luciferase reporter assay

The candidate target sequences of LINC00922 and HMGA2 were inserted into pGL3-basic vector (Promega, USA) to construct LINC00922 wild-type (LINC00922-WT) or LINC00922 mutant (LINC00922-MUT) or HMGA2-WT or HMGA2-MUT separately. The binding sites of 3′-UTR of LINC00922 were mutated from 5′-UGGCAUAGGAACAAAGGGAAC-3′ to 5′-UCCGUAUCCUAGUUUCCCUUC-3′ for miR-204-5p, and the potential binding sites of HMGA2 were mutated from 5′-AGGGUGGGGGGAAGAGGAGGGGAA-3′ to 5′-AUAACAGGAGAAAUCGAUAAAUUC-3′ for miR-204-5p. Cells were seeded into a 24-well plate and co-transfected with constructed luciferase plasmids and miR-204-5p mimic or inhibitor for 48 h. Luciferase motility was detected by Dual-Luciferase Reporter Assay System (Promega, USA).

### RNA immunoprecipitation (RIP) assay

RIP analysis was performed using RIP kit (Millipore, USA). In short, the collected cells were centrifuged at 2500×*g* for 15 min to pellet the nuclei. The nuclear pellet was then resuspended in RIP buffer for 30 min. The cell extracts were incubated with protein A/G sepharose beads coupled with antibodies against Ago2 for 2 h. Total RNA from whole cell lysates was analyzed by qRT-PCR.

### Western blot assay

Total protein was isolated with RIPA and quantified by BCA assay kit (Beyotime, China). The same amount of protein was placed by SDS-PAGE, and then shifted it to the PVDF membrane. Then, the membrane was incubated with the primary antibody (anti-HMGA2, Ab207301,1:1000, Abcam, USA; anti-GAPDH, ab9485, 1:5000, Abcam, USA) at 4 °C for 12 h, and the secondary antibody for 1 h. Finally, ECL kit (ThermoFisher, USA) was used to visualize the membrane.

### Tumor xenograft model

Animal care and euthanasia were carried out with the approval by the Ethics Committee of The First Affiliated Hospital of Soochow University. 4–6-week-old nude mice were divided into two groups: sh-NC group (injection of HGC-27 cells with sh-NC), sh-LINC00922 group (injection of HGC-27 cells with sh-NC). In brief, 200 μL HGC-27 cell suspension (1 × 10^7^ cells) was subcutaneously injected into the mice. After four weeks, mice were sacrificed with 100 mg/kg sodium pentobarbital. The gastric tumors were dissected, photographed, and weighted.

### Hematoxylin and eosin (HE) staining

The gastric tumors were fixed in 4% paraformaldehyde (Sigma, USA) 48 h, and embedded in paraffin. Thereafter, tissues were sliced and the sections were dyed with hematoxylin for 2 min and eosin for 1 min. Images were viewed by a light microscope (Nikon, Japan).

### Immunohistochemistry (IHC) staining

The sections of gastric tumors were incubated with anti-Ki67 (ab16667, 1:200, Abcam, USA) at 4 °C for 12 h and secondary antibody at room temperature for 30 min. At last, the sections were visualized with DAB (Abcam, USA) for 1 min. Images were viewed by a light microscope (Nikon, Japan).

### TUNEL staining

TUNEL staining was performed using TUNEL detection kit (Beyotime, China). The sections of gastric tumors were incubated with proteinase K for 15 min and 3% H2O2 for 20 min. Then incubated with 50 μl Streptavidin-HRP for 30 min and immersed by DAB. Images were viewed by a light microscope (Nikon, Japan).

### Statistical analysis

All experiments were independently repeated three times. The data were presented as the mean ± standard deviation (SD). SPSS (18.0 version) and GraphPad (6.0 version) were used to determine each group statistic data. Moreover, Student’s *t* test and one-way ANOVA were used to compare two or multiple groups.

## Results

### LINC00922 is up-regulated in GC tissues and cell lines

**Fig. 1:**
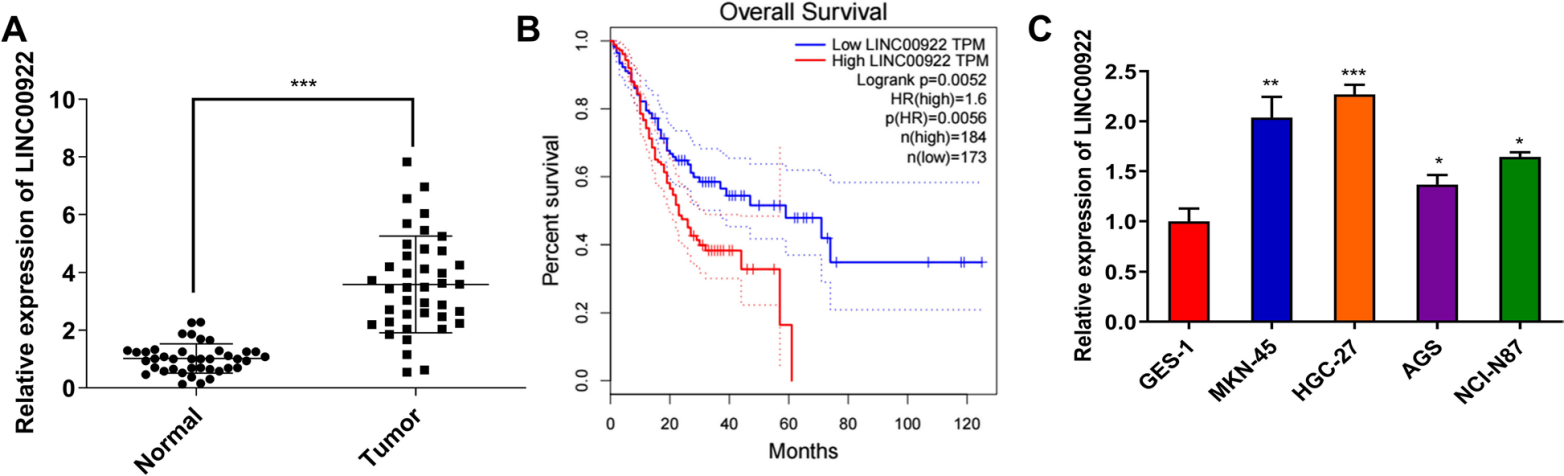
LINC00922 is upregulated in GC tissues and cell lines. **A** LINC00922 level in 40 pairs of GC tissues and adjacent normal tissues was verified by qRT-PCR assay. ***P < 0.001 *vs*. Normal group. **B** Kaplan-Meier analysis assessed the survival curves in GC patients based on LINC00922 expression. **C** LINC00922 level in GC cell lines (MKN-45, HGC-27, AGS, NCI-N87) and epithelial cell line (GES-1) was verified by qRT-PCR assay. **P < 0.01, ***P < 0.001 *vs*. GES-1 group

To identify the level of LINC00922 in GC, qRT-PCR assay was firstly employed in GC samples. The abundance of LINC00922 in GC tissues was higher (mean ratio of 3.59-fold) than adjacent tissues (Fig. 1A). Besides, LINC00922 was associated with poor prognosis and survival time in patients with GC (Fig. 1B). Consistent with clinical samples, LINC00922 level was markedly increased in MKN-45, HGC-27, AGS, NCI-N87 compared with GES-1 (Fig. 1C). As LINC00922 expression in MKN-45, HGC-27 had a higher ratio (2.04-fold and 2.27-fold, respectively), MKN-45 and HGC-27 were chosen for the next experiments (Fig. 1C). Above all, LINC00922 expression is increased in GC, which indicates that upregulation of LINC00922 may exert a function in GC progression.

### Inhibition of LINC00922 suppresses GC progression in vitro

**Fig. 2:**
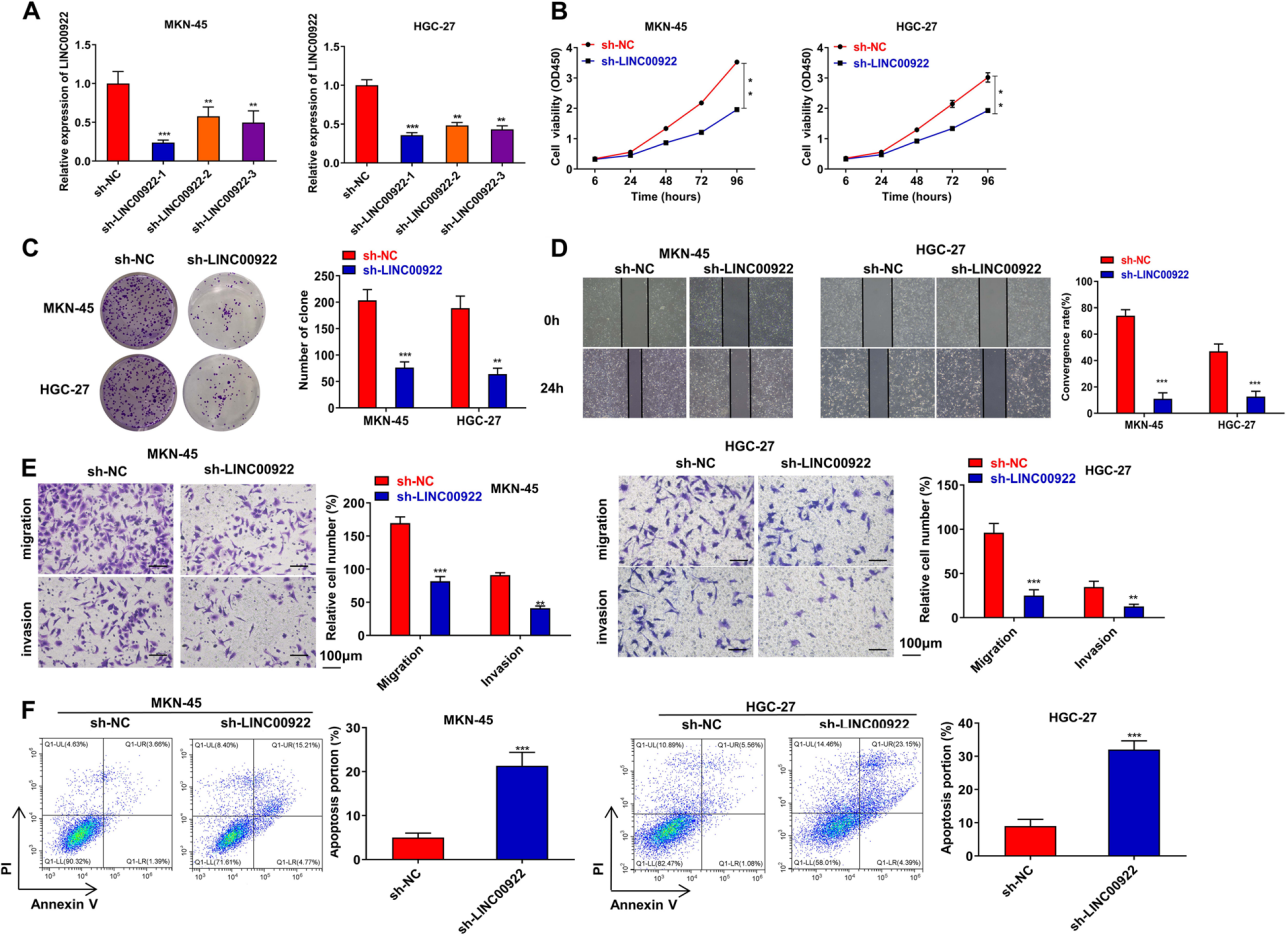
Inhibition of LINC00922 suppresses GC progression in vitro. Sh-NC and sh-LINC00922 were transfected to MKN-45 and HGC-27. **A** The transfection efficiency was certified by qRT-PCR assay. **B** CCK-8 and **C** colony formation assays were performed to investigate the proliferation of LINC00922. **D** Wounding healing and **E** transwell assays were used to investigate the migration and invasion of LINC00922. **F** Flow cytometry assay was used to assess cell apoptosis of LINC00922. *P <0.05, **P < 0.01, ***P < 0.001 *vs.* sh-NC group

To verify the exact roles of LINC00922 in gastric progression, sh-LINC00922 sequences were transfected to knock down LINC00922 in MKN-45 and HGC-27. The transfection effect of sh-LINC00922 was showed in Fig. 2A. As sh-LINC00922-1 group had a lower ratio of LINC00922 (0.24-fold and 0.35-fold, respectively) in MKN-45, HGC-27, sh-LINC00922-1 was selected for the further experiment (Fig. 2A). Inhibition of LINC00922 dramatically suppressed the cell viability and the number of colonies in MKN-45 and HGC-27 (Fig. 2B, C). Consistently, wounding healing and transwell assays clarified that inhibition of LINC00922 reduced cell migration and invasion (Fig. 2D–E). Furthermore, flow cytometry assay displayed that cell apoptotic rate was markedly elevated by knockdown of LINC00922 (Fig. 2F). These results demonstrate that suppression of LINC00922 reduces cell proliferation, migration, invasion, and activates cell apoptosis in GC.

### LINC00922 targets miR-204-5p in GC

**Fig. 3:**
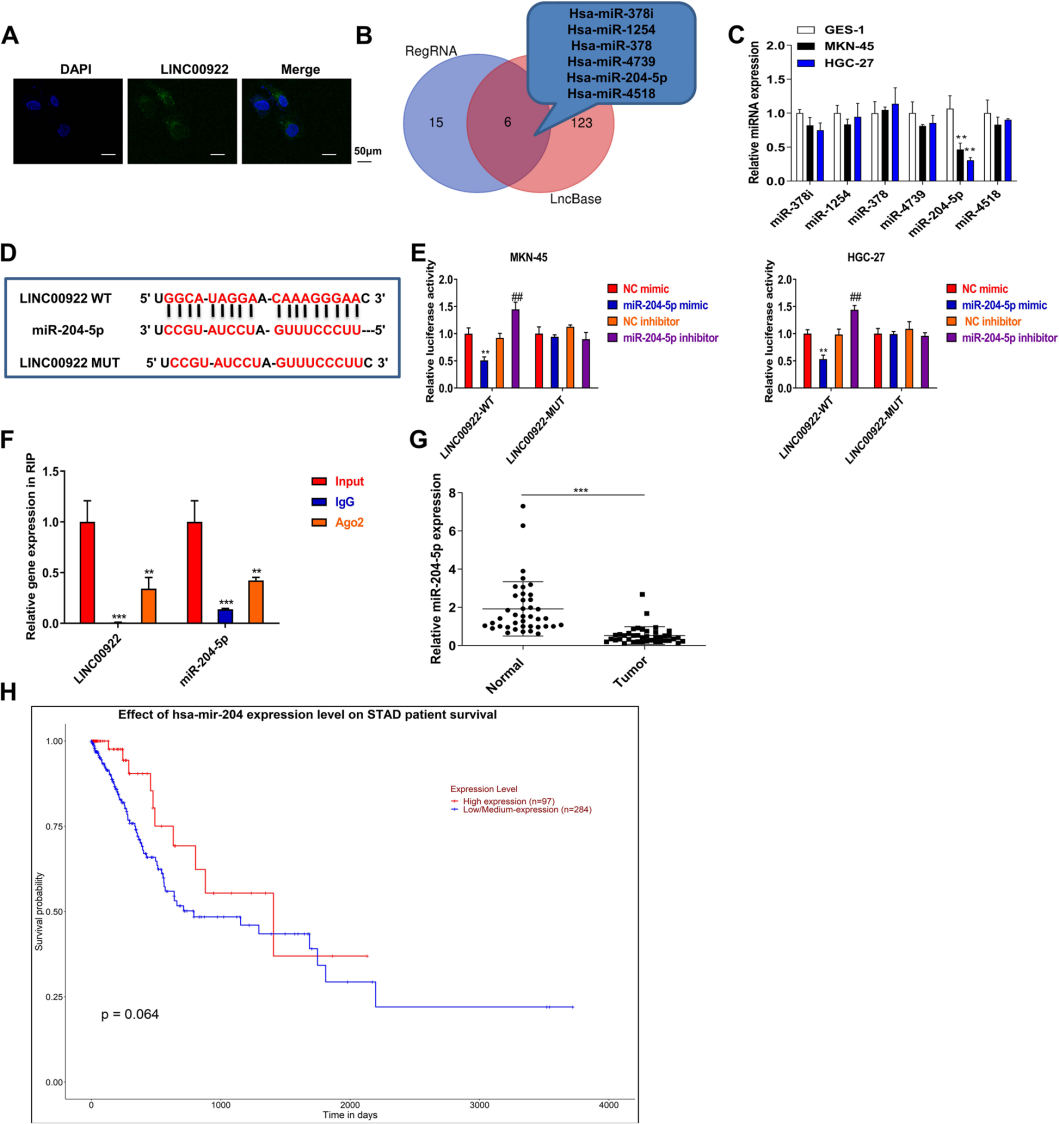
LINC00922 targets miR-204-5p in GC. **A** FISH analysis was performed to analyze LINC00922 in either the cytoplasm or nucleus. **B** The online databases RegRNA 2.0 and LncBase v.2 were used to predict possible miRNAs. **C** The expression of miRNAs was measured by qRT-PCR assay in MKN-45 and HGC-27, GES-1. *P < 0.05, **P < 0.01 *vs*. GES-1 group. **D** The potential binding sites of miR-204-5p with 3′UTR of LINC00922. **E** Luciferase reporter assay and **F** RNA pull-down assays were used to confirm the interaction between LINC00922 and miR-204-5p. **P < 0.01 *vs*. NC mimic group, ## *P* < 0.01 *vs*. NC inhibitor group. **G** miR-204-5p level in 40 pairs of GC tissues and adjacent normal tissues was verified by qRT-PCR assay. **P < 0.01 *vs*. Normal group. **H** Kaplan-Meier analysis assessed the survival curves in GC patients based on miR-204-5p expression

As ceRNAs, lncRNAs bind to miRNAs and act as miRNA sponges in GC [25]. FISH analysis revealed LINC00922 was markedly located in the cytoplasm, indicating LINC00922 might serve as a ceRNA in GC (Fig. 3A). The online databases RegRNA 2.0 and LncBase v.2 were used to predict possible miRNAs (Fig. 3B). Besides, RegRNA 2.0 screening conditions were score ≥ 150 and free energy ≤ − 21 (Fig. 3B). After screening, miR-378i, miR-1254, miR-378, miR-4739, miR-204-5p, and miR-4518 were found to be common miRNAs in the databases (Fig. 3B). Then, we found miR-204-5p was decreased in MKN-45 and HGC-27 (0.46-fold and 0.31-fold, respectively) compared with GES-1 (Fig. 3C). Hence, miR-204-5p was chosen for predicting potential miRNA associated with LINC00922. The potential binding sites of miR-204-5p with 3′UTR of LINC00922 were shown in Fig. 3D. MiR-204-5p decreased the luciferase activity of the LINC00922 WT rather than LINC00922 WT, indicating that miR-204-5p specifically bound to the 3′-UTR of LINC00922 (Fig. 3E). RIP assay confirmed the interaction between LINC00922 and miR-204-5p (Fig. 3F). Furthermore, the abundance of miR-204-5p in GC tissues was markedly lower than adjacent tissues, among which downregulated miR-204-5p associated with poor prognosis in GC (Fig. 3G, H). According to the above data, LINC00922 acts as a sponge of miR-204-5p in GC.

### MiR-204-5p suppresses GC progression in vitro

**Fig. 4:**
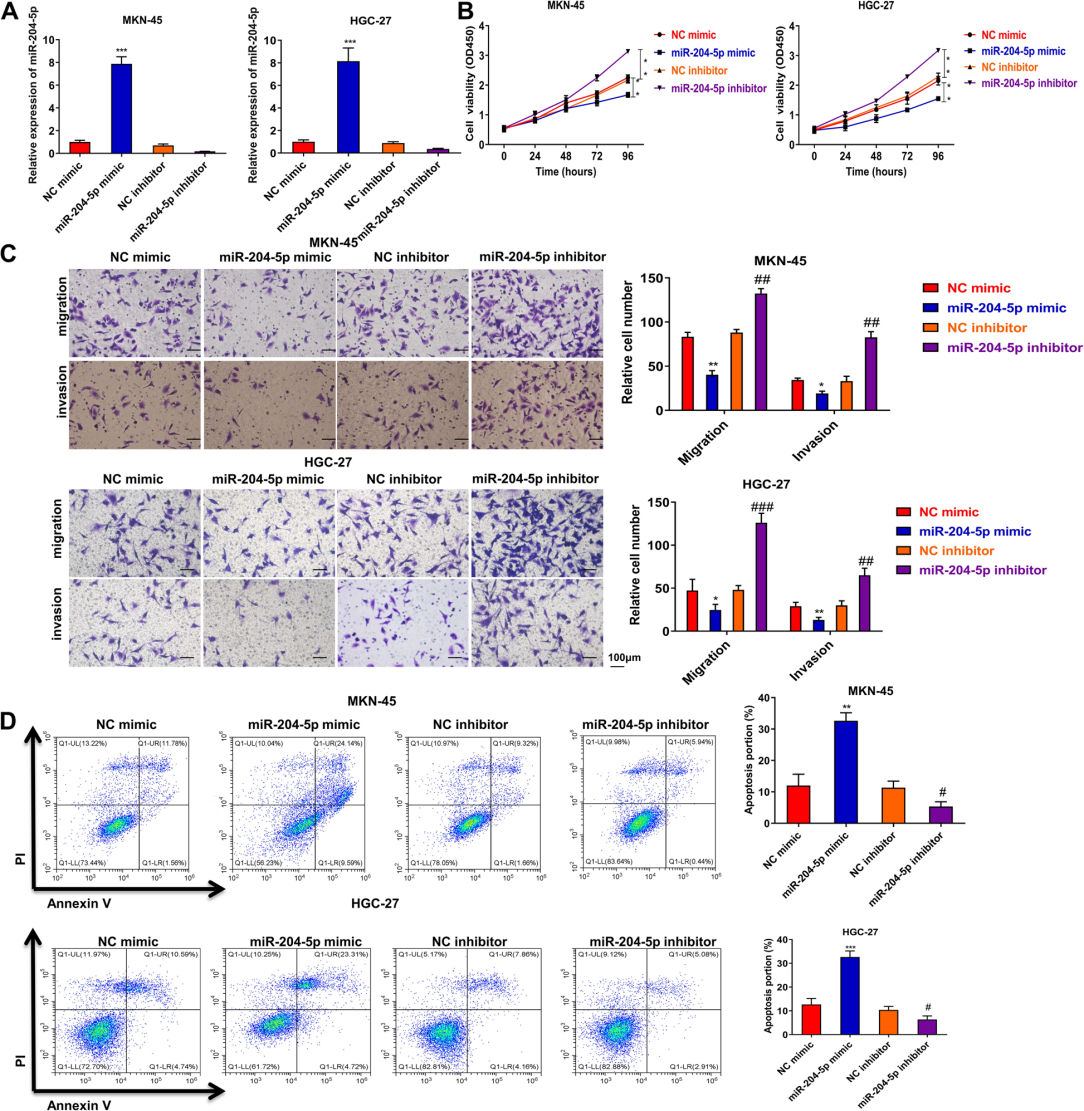
MiR-204-5p suppresses GC progression in vitro. MiR-204-5p mimic or inhibitor was transfected to MKN-45 and HGC-27. **A** The transfection efficiency was certified by qRT-PCR assay. **B** CCK-8 assay was performed to investigate the proliferation. **C** Transwell assay was used to investigate the migration and invasion. **D** Flow cytometry assay was used to assess cell apoptosis. *P < 0.05, **P < 0.01, ***P < 0.001 *vs.* NC mimic group, ##*P* < 0.01, ### *P* < 0.001 *vs*. NC inhibitor group

To further explore the role of miR-204-5p in gastric progression, miR-204-5p mimic and inhibitor were transfected to upregulate or downregulate miR-204-5p in MKN-45 and HGC-27. The transfection effects of miR-204-5p mimic and inhibitor were showed in Fig. 4A. Then, CCK-8 assay indicated that miR-204-5p mimic dramatically suppressed the cell viability, while miR-204-5p inhibitor notably promoted the cell viability of MKN-45 and HGC-27 (Fig. 4B). Consistently, transwell assay clarified that miR-204-5p mimic remarkably reduced cell migration and invasion, while miR-204-5p inhibitor had opposite effects (Fig. 4C). Moreover, miR-204-5p mimic accelerated cell apoptosis, while miR-204-5p inhibitor notably decreased the apoptosis (Fig. 4D). Taken together, these results clarify that miR-204-5p reduces GC progression.

### MiR-204-5p targets HMGA2 in GC

**Fig. 5:**
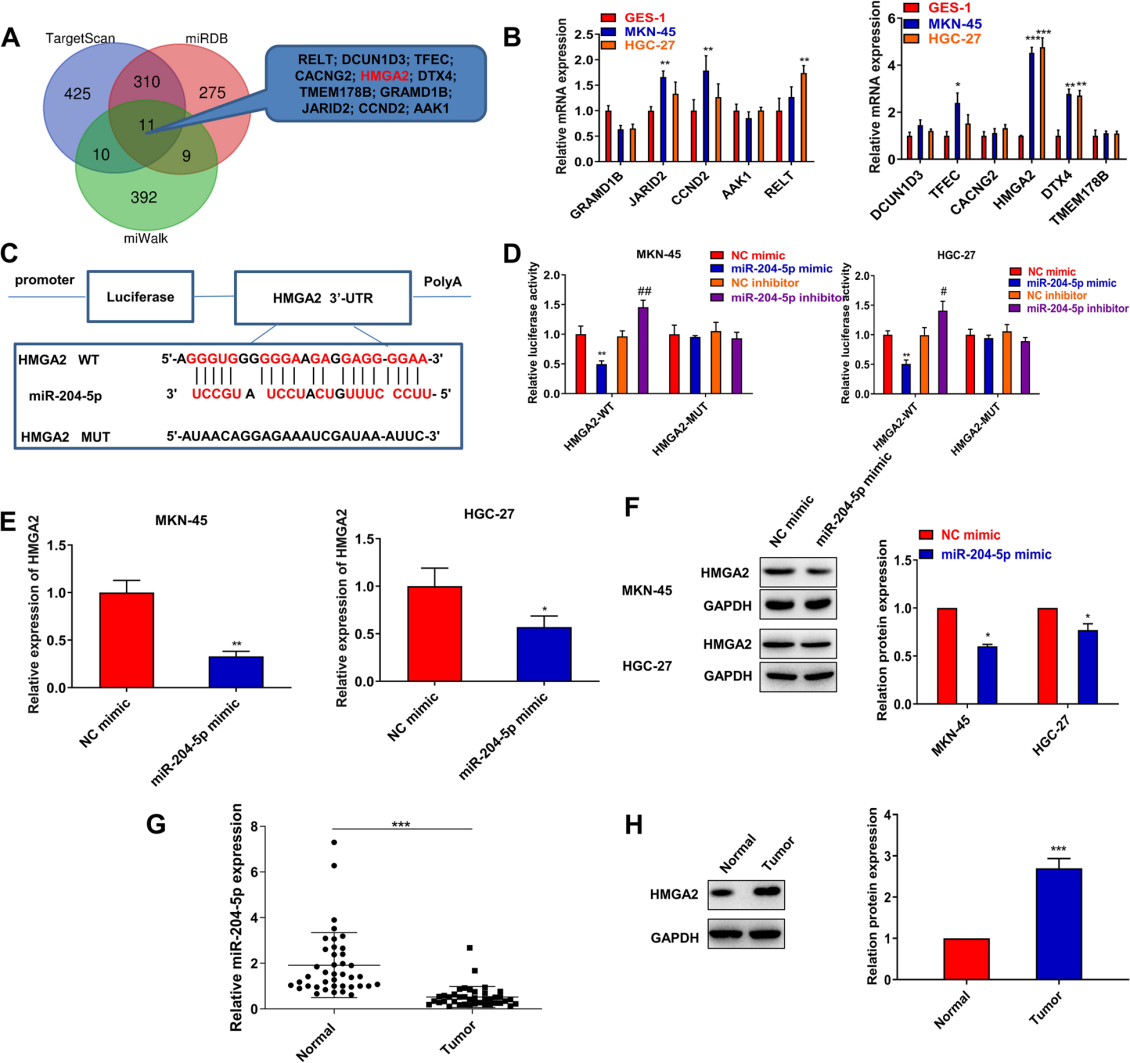
MiR-204-5p targets HMGA2 in GC. **A** Three online databases (Targetscan 5.0, miRDB, and miRWalk 2.0) to predict common targets of miR-204-5p. **B** The levels of targets were measured by qRT-PCR assay in MKN-45, HGC-27, and GES-1. *P < 0.05, **P < 0.01, ***P < 0.001 *vs*. GES-1 group. **C** The potential binding sites of miR-204-5p with 3′UTR of HMGA2. **D** Luciferase reporter assay was used to assess the interaction between miR-204-5p and HMGA2. **P < 0.01 *vs*. NC mimic group, ## *P* < 0.01 *vs*. NC inhibitor group. **E** qRT-PCR and **F** western blot assays were used to assess HMGA2 level in MKN-45 and HGC-27 cells. **P < 0.01 *vs*. NC mimic group. **G** qRT-PCR was used to assess HMGA2 mRNA level in GC tissues 40 pairs of GC tissues and adjacent normal tissues. **H** Western blot assays were used to assess HMGA2 protein level in GC tissues. ***P < 0.001 *vs*. Normal group

To find the putative targets of miR-204-5p, we performed three online databases (Targetscan 5.0, miRDB, and miRWalk 2.0) to predict common targets (Fig. 5A). Besides, miRDB screening conditions were energy ≤ − 25, and miRDB screening conditions were Targetscore ≥ 50. After screening, we finally found 11 intersecting genes of miR-204-5p (Fig. 5A). Additionally, we checked the expression of 11 intersecting target genes in GC cell lines. The result showed that HMGA2 expression had a higher ratio in MKN-45, HGC-27 (4.52-fold and 4.76-fold, respectively) (Fig. 5B). Therefore, HMGA2 was chosen as a candidate gene for further study. We further constructed a luciferase reporter gene that mutated the putative miR-204-5p binding site in the HMGA2 sequence (Fig. 5C). We found that miR-204-5p was specifically bound to HMGA2 in GC (Fig. 5D). Besides, miR-204-5p negatively regulated HMGA2 level as indicated by qRT-PCR and western blot assays (Fig. 5E, F). We also found HMGA2 level was increased in the GC tissues (Fig. 5G, H). In short, these results demonstrate that HMGA2 is a target of miR-204-5p.

### Inhibition of LINC00922 suppresses GC progression via targeting miR-204-5p /HMGA2 axis in vitro

**Fig. 6:**
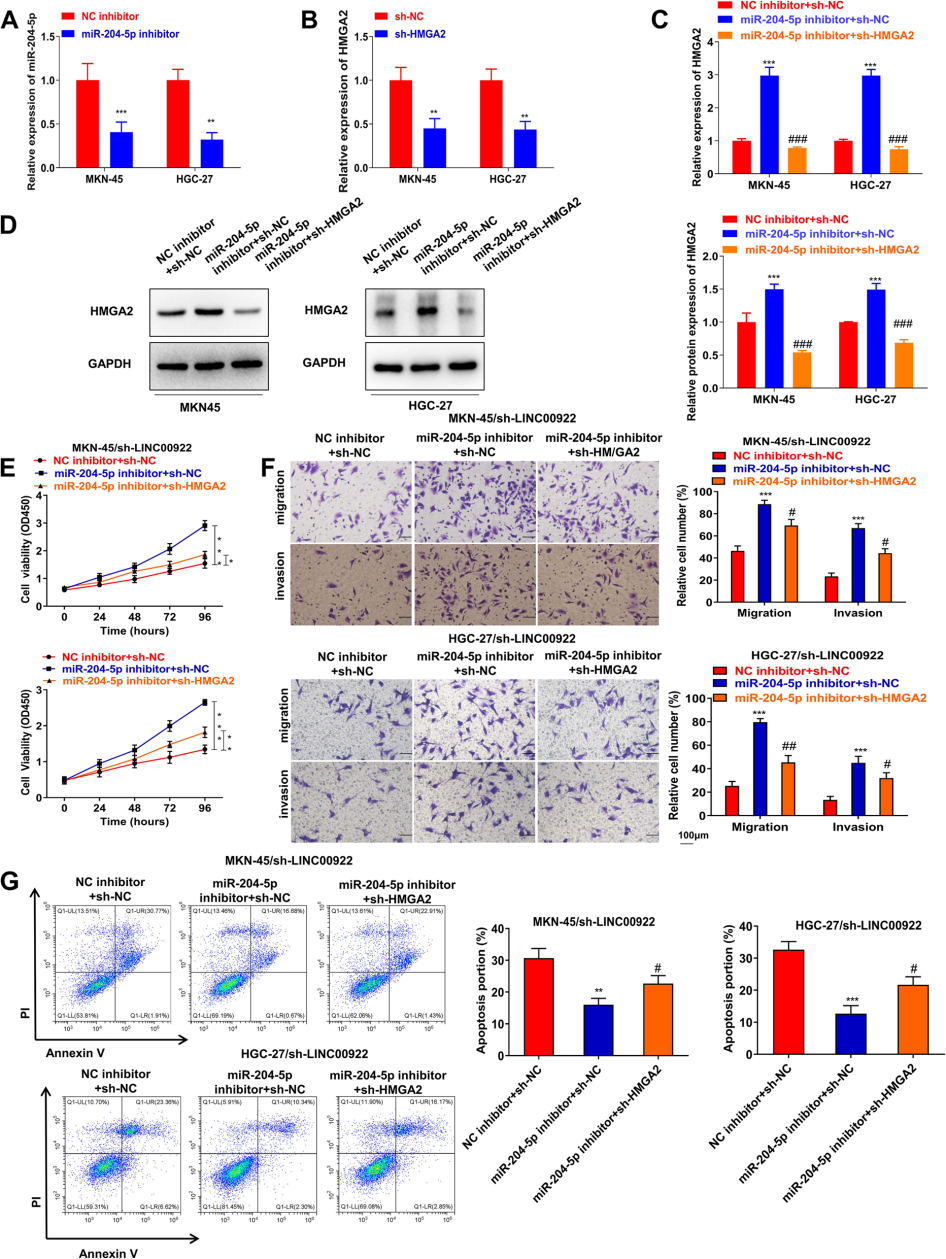
Inhibition of LINC00922 suppresses GC progression via targeting miR-204-5p /HMGA2 axis *in vitro.* (A) The transfection efficiency of miR-204-5p inhibitor was certified by qRT-PCR assay. *P < 0.05, **P < 0.01, ***P < 0.001 *vs*. NC inhibitor group. **B** The transfection efficiency of sh-HMGA2 was certified by qRT-PCR assay. *P < 0.05, **P < 0.01, ***P < 0.001 *vs*. sh-NC group. **C**, **D** qRT-PCR and western blot assays were used to assess HMGA2 expression in MKN-45 and HGC-27 cells with miR-204-5p inhibitor and/or sh-HMGA2 transfection. MKN-45/sh-LINC00922 and HGC-27/sh-LINC00922 cells were co-transfected with miR-204-5p inhibitor and/or sh-HMGA2. **E** CCK-8 assay was performed to investigate the proliferation. **F** Transwell assay was used to investigate the migration and invasion. **G** Flow cytometry assay was used to assess cell apoptosis. **P < 0.01, ***P < 0.001 *vs.* NC inhibitor + sh-NC group, #*P* < 0.05, ## *P* < 0.01 *vs*. miR-204-5p inhibitor + sh-NC group

To explore how LINC00922 influenced its downstream targets in the progression of GC, MKN-45/sh-LINC00922 and HGC-27/sh-LINC00922 cells co-transfected with miR-204-5p inhibitor and/or sh-HMGA2. MiR-204-5p and HMGA2 levels were downregulated by application of miR-204-5p inhibitor and sh-HMGA2 (Fig. 6A, B). qRT-PCR and western blot assays showed that sh-HMGA2 reversed miR-204-5p inhibitor-induced HMGA2 upregulation (Fig. 6C, D). The decreased proliferation, migration, invasion of MKN-45/sh-LINC00922 and HGC-27/sh-LINC00922 cells were reversed by miR-204-5p inhibitor and therewith recovered due to suppression of HMGA2 (Fig. 6E, F). Besides, MKN-45/sh-LINC00922 and HGC-27/sh-LINC00922 cells co-transfected with miR-204-5p inhibitor partially decreased cell apoptosis, while further downregulation of HMGA2 reversed this effect (Fig. 6G). Therefore, LINC00922 functions as a facilitator in GC through targeting miR-204-5p/HMGA2 axis.

### Inhibition of LINC00922 suppresses GC progression in vivo

**Fig. 7:**
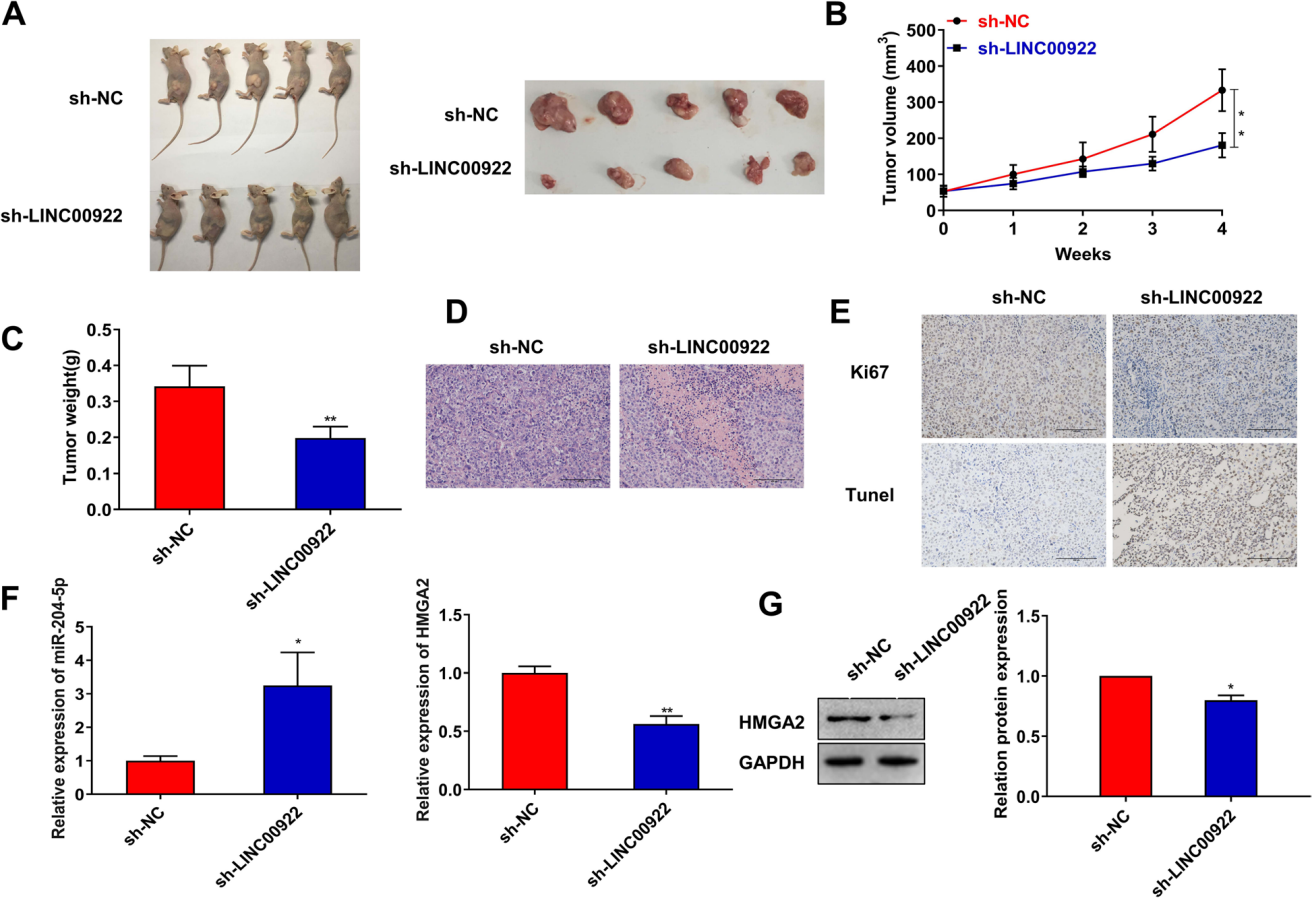
Inhibition of LINC00922 suppresses GC progression in vivo. Nude mice were subcutaneously inoculated with HGC-27 cells with sh-NC or sh-LINC00922 transfection. **A** Representative images of GC tumors (*n* = 5). **B** Tumor volume and **C** tumor weight of the xenograft were measured. **D** HE staining was conducted to observe tumor infiltration. **E** IHC and TUNEL staining were used to assess Ki67 expression and cell apoptosis. **F** qRT-PCR was used to assess miR-204-5p and HMGA2 mRNA levels in GC tissues. **G** Western blot assays were used to assess HMGA2 protein level in GC tissues. *P < 0.05, **P < 0.01 *vs*. sh-NC group

To further evaluate the therapeutic potential of LINC00922 in vivo, we implemented the xenograft model in mice. As similar to the results of the in vitro study, knockdown of LINC00922 repressed the tumorigenicity of HGC-27 cells, and repressed the tumor growth and weight (Fig. 7A–C). In addition, HE staining suggested knockdown of LINC00922 inhibited tumor infiltration (Fig. 7D). IHC and TUNEL staining showed knockdown of LINC00922 suppressed the expression of the proliferating protein Ki67, and led to cell apoptosis (Fig. 7E). Besides, knockdown of LINC00922 promoted miR-204-5p level, and decreased HMGA2 level (Fig. 7F, G). In conclusion, these findings unravel that LINC00922 accelerated the deterioration of GC.

## Discussion

Recently, although great advances have been made in clinical therapeutic approaches, the survival rate of GC patients is still very low [26]. Therefore, it is urgent to search for novel therapeutic targets for GC. In recent years, lncRNAs have attracted immense research interests worldwide [27, 28]. In the current study, we found that LINC00922 play oncogenic effects through sponging for miR-204-5p, leading to the upregulation of HMGA2.

Emerging studies have declared that lncRNAs participated in the pathogenesis of GC [29]. Here, we identified the upregulated LINC00922 in GC. Aberrant expression of LINC00922 has been revealed to be associated with cancers. For instance, previous studies suggested that upregulation of LINC00922 promoted lung cancer cell proliferation [16]. Besides, abnormal expression of LINC00922 promoted epithelial-mesenchymal transformation and the occurrence of breast cancer by Wnt pathway [17]. Additionally, Gu Z et al. suggested that LINC00922 regulated the progression of doxorubicin-resistant osteosarcoma [15]. Here, the functional role of LINC00922 was investigated in GC cells and xenograft model. Our data revealed that inhibition of suppressed GC progression in vitro and in vivo, indicating LINC00922 could be an oncogene in GC.

Intracellular localization of lncRNA is the basis of its function [30]. LncRNAs located in the nucleus participate in epigenetic modification or transcriptional regulation [31, 32]. However, lncRNAs located in cytoplasm are involved in post-transcriptional regulation mainly by affecting the stability of mRNA molecules. Our data revealed that LINC00922 was markedly located in the cytoplasm, indicating LINC00922 might serve as a ceRNA in GC. Besides, we identified miR-204-5p was a target of LINC00922 in GC. In the present study, we found miR-204-5p negatively correlated with gastric cell growth. These results were consistent with Chen X [24], Liang Y [33]. Liang Y et al. suggested that miR-204-5p contributed to the activation of GC through inhibiting organic cation transporter 1 (OCT1) expression level [33]. In addition, miR-204-5p has been proved to have important biological functions in many diseases, such as osteoarthritis [34], prostate cancer [35], and cervical cancer [36].

It is accepted that miRNAs bind to the 3′-UTR complementary nucleotide sequence of target gene, thereby inhibiting the translation or degradation of mRNA and realizing the post-transcriptional regulation of genes [37]. In the present study, we found that miR-204-5p negatively regulated the expressions of HMGA2 by directly targeting its 3′-UTR. HMGA2 is considered as an oncogene and is involved in many carcinogenic signaling networks as p53, and Wnt/β-catenin signaling pathways [38–40]. Here, HMGA2 level was upregulated in GC. Moreover, the rescue experiment found that down-regulation of HMGA2 partially reversed the oncogenic role caused by miR-204-5p inhibitor.

Liang T et al. revealed that miR-204 reversed the promotive effects of LINC00922 on the cellular behaviors of lung cancer, and provided a link between LINC00922 and miR-204 in lung cancer [16]. However, the roles of LINC00922/miR-204-5p axis on GC progression remain unknown. In the present study, the online databases RegRNA 2.0 and LncBase v.2 showed that miR-204-5p might be bound to LINC00922, and miR-204-5p was downregulated in GC. The Functional experiments indicated that LINC00922 promoted GC progression via targeting miR-204-5p. Given the feature that lncRNA binds to multiple miRNAs to participate in cancers. Therefore, it is worthwhile to add considerable effort to reveal additional miRNAs that are potentially bound to LINC00922 during GC progression.

## Conclusions

We firstly identified the participation of LINC00922 in GC and in-depth study the mechanism of LINC00922. LINC00922 functions as GC-promoting ceRNA via targeting miR-204-5p/HMGA2 axis. The LINC00922/miR-204-5p/HMGA2 axis might provide novel insight for GC-targeted therapy.

## Supplementary Information

**Additional file 1.**

## Data Availability

The data used to support the findings of this study are available from the corresponding author upon request.
