# Supplementary material for: LINC00922 acts as a novel oncogene in gastric cancer
Source: World J Surg Oncol. 2022 Apr 15;20:121. doi: 10.1186/s12957-022-02569-3 (PMC9013058; doi:10.1186/s12957-022-02569-3)
Supplement: Supplementary file 1 — Additional file 1. [file 12957_2022_2569_MOESM1_ESM.docx]

**Supplementary table 1 Characteristics of participants**

| Parameters | Total 40 (100%) |
| --- | --- |
| **Age** |  |
| ≥60 | 29 (72.50%) |
| <60 | 11 (27.50%) |
| **Gender** |  |
| Female | 25 (62.50%) |
| Male | 15 (37.50%) |
| **Tumor size (cm)** |  |
| <4 | 12 (30.00%) |
| ≥4 | 28 (70.00%) |
| **Pathological stage** |  |
| I+II | 10 (25.00%) |
| III+VI | 30 (75.00%) |
| **Lymphatic invasion** |  |
| Positive | 9 (22.50%) |
| Negative | 31 (77.50%) |

**Supplementary table 2 The sequences for transfection**

| **Gene** | **Sequences** (5′-3′) |
| --- | --- |
| sh-LINC00922-1 | CCGGCCTGCACCTACAGATCTACACCTCGAGGTGTAGATCTGTAGGTGCAGGTTTTTG |
| sh-LINC00922-2 | CCGGGAACCTACATTCACAACATAACTCGAGTTATGTTGTGAATGTAGGTTCTTTTTG |
| sh-LINC00922-3 | CCGGTGCAGGAAGTGTTCATCTAAGCTCGAGCTTAGATGAACACTTCCTGCATTTTTG |
| sh-HMGA2 | CCGGCCAACCTTCAAAGAACTTGAACTCGAGTTCAAGTTCTTTGAAGGTTGGTTTTT |
| sh-NC | CCGGCAACAAGATGAAGAGCACCAACTCGAGTTGGTGCTCTTCATCTTGTTGTTTTT |
| miR-204-5p mimic | UUCCCUUUGUCAUCCUAUGCCU |
| miR-204-5p inhibitor | AGGCAUAGGAUGACAAAGGGAA |
| NC mimic | UUUGUACUACACAAAAGUACUG |
| NC inhibitor | CAGUCCUUUUGUGUAGUACAA |

**Supplementary table 3 Primer sequences of genes in RT-qPCR assay**

| **Gene** | **Forward Primer (**5′-3′) | **Reversed Primer (**5′-3′) |
| --- | --- | --- |
| LINC00922 | AAGGCTCGACTCTCTGTCCT | GTCTTTGCCTGTTTCAGCGG |
| miR-378i | GCGGCGGACTGGACTAGGAGTC | CGCACTGGATACGACCAGCTG |
| miR-1254 | AGCCTGGAAGCTGGAGCCTGCAGT | GCGAGCACAGAATTAATACGAC |
| miR-378 | CCTGACTCCAGGTCCT | GAACATGTCTGCGTATCTC |
| miR-4739 | GCTGGGACATTGAAAGTCTCA | GATGTTCCCATCGGCGTGTC |
| miR-204-5p | ACACTCCAGCTGGGTTCCCTTTGTCATCCTAT | TGTCATCCTAT |
| miR-4518 | GGAGGGATGATAACTGTGCTGAGA | TCAATCCCA GCTCTTTT |
| RELT | CTGTCTTCTGCCTCATGGGG | TGGTGTCCTCATTGGCATCC |
| DCUN1D3 | CTCCTGTTCCCAGCCTTCTG | GCCCACAGACAAGATGGTGA |
| TFEC | TTAGAGCAGGCTAACAGGCG | ATGGCTCTGCTGTTTGGTGA |
| CACNG2 | TATCTGCCAATGCCGGAGAC | GCCGGTCGATAAACATGTGC |
| HMGA2 | GCAGACCTAGGAAATGGCCA | AGTCCTCTTCGGCAGACTCT |
| DTX4 | TCAACACCATGGGCCAGATC | CCTTAGGCAAGGTCCCTGTG |
| TMEM178B | CAACCATCCCCAGGAACCTC | ACGTACTGCATAAGGCCTCG |
| GRAMD1B | TGACTCTTCCGACACACACG | GAAGGGCGAGTTGGTGAAGA |
| JARID2 | ATTGCTACCCCCTCCAGCTA | CCTCCTCGACTTCCTCCTCA |
| CCND2 | TACCTGGACCGTTTCTTGGC | CGGGCTGGTCTCTTTGAGTT |
| AAK1 | CGTAACCCACAGTGCAGTCT | GATGGAGTGGTGGTTGCAGA |
| GAPDH | GAGAAGGCTGGGGCTCATTT | AGTGATGGCATGGACTGTGG |
| U6 | CTCGCTTCGGCAGCACA | AACGCTTCACGAATTTGCGT |
